# Geographic patterns of insect diversity across China's nature reserves: The roles of niche conservatism and range overlapping

**DOI:** 10.1002/ece3.6097

**Published:** 2020-03-14

**Authors:** Yueming Lyu, Xiangping Wang, Juchun Luo

**Affiliations:** ^1^ College of Forestry Beijing Forestry University Beijing China

**Keywords:** area, contemporary climate, habitat heterogeneity, historical processes, niche conservatism, phylogenetic structure, range overlapping

## Abstract

**Aim:**

Insects are the most species‐rich clade in the world, but the broad‐scale diversity pattern and the potential drivers have not been well documented for the clade as a whole. We aimed to examine the relative roles of contemporary and historical climate, niche conservatism, range overlapping, and other environmental factors on geographic patterns of species richness and phylogenetic structure, for insects across China.

**Location:**

China.

**Methods:**

We collected insect data from 184 nature reserves and examined geographic patterns of species richness and mean root distance (MRD, a metric of the evolutionary development of assemblages) for different biogeographic affinities (Palearctic, Oriental, and widespread species) and for clades originated during the warm and cold geohistorical periods (“warm clades” and “cold clades,” respectively). We related richness and MRD to contemporary and historical climate, area, habitat heterogeneity, and human disturbance to evaluate their relative importance.

**Results:**

Total species richness revealed a hump‐shaped latitudinal pattern, peaking between 30°~35°N. Richness patterns differed markedly among evolutionary groups: Oriental species richness decreased significantly with higher latitude but Palearctic species increased, while other groups again peaked between 30°~35°N. The range overlapping of different biogeographic groups in midlatitudes may be an important contributor to humped latitudinal richness patterns. MRD was positively related to latitude and increased more rapidly for “warm clades” than “cold clades.” Historical climate factors (especially winter coldness) were among the strongest predictors for both richness and phylogenetic patterns, for each evolutionary group, suggesting the strong influence of niche conservatism.

**Conclusions:**

The hump‐shaped latitudinal pattern of insect richness in China is mainly shaped by niche conservatism and range overlapping, supplemented by habitat heterogeneity and contemporary climate. The role of niche conservatism and range overlapping may have been overlooked if only total species richness was analyzed, suggesting the importance of examining different evolutionary groups separately.

## Graphical abstract

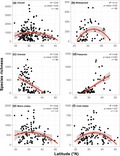

## 1 INTRODUCTION

Species richness is generally reported to peak at the tropics and decline as latitude increases in the northern and southern hemisphere, for a number of taxa (Chown & Gaston, 2000). This latitudinal diversity gradient (LDG hereafter) has long been a theoretical focus in macroecology and biogeography since it was first observed by von Humboldt in 1874. However, studies have found many exceptions to this “classical LDG,” where species richness showed increased, humped, or null pattern with higher latitude for some taxa (snake: Pyron & Burbrink, 2009; fish: Rabosky et al., 2018). For example, a recent study on global grasses LDG showed that the richness of Poaceae was lower in the tropics and higher in the midlatitudes of both northern and southern hemispheres (Visser et al., 2014). Smith et al. (2005) also found that tree frogs in the northern hemisphere revealed the highest diversity in the midlatitudes of each continent. For some mammal and bird clades, more species occupied at cooler temperatures, or species richness peaked at midlatitudes (Buckley et al., 2010; Ding, Yuan, Geng, Koh, & Lee, 2006). Even in the soil, some microorganisms and animals’ species richness may peak in midlatitudes (Bahram et al., 2018; Fernández et al., 2016). Recent studies have increasingly suggested the value of these “exceptions” in understanding the drivers of LDG (Jansson & Davies, 2008; Visser et al., 2014). For instance, if the current hypotheses on LDG (see below) are really general ones, they should be able to explain not only the “classical” LDG but also the “exceptional” latitudinal patterns.

For more than a century, ecologists have made great efforts to explore the underlying mechanisms of geographic diversity patterns and many hypotheses have been proposed (Rahbek et al., 2007). The earlier hypotheses were based on the widely observed close relationship between diversity and contemporary climate, and suggested energy and water availability (or productivity) as major drivers (e.g., Hawkins & Porter, 2003; O'Brien, 1993). In addition to these explanations based on ecological processes, some authors proposed alternative hypotheses based on regional processes, which suggests that evolutionary history is critical in shaping not only the global diversity patterns but also the relationship between diversity and contemporary climate gradient (e.g., Ricklefs, 1987, 2005). In recent years, with the rapid progress of molecular and biogeographic techniques, the influence of historical processes has been increasingly accepted. The tropical niche conservatism hypothesis proposed that (Wiens & Donoghue, 2004): Many clades have a tropical origin and cannot colonize colder areas because they have maintained their warm‐weather affinities as a result of niche conservatism (or phylogenetic inertia). Because the evolution of cold tolerance is evolutionarily costly, only a subset of clades has managed to spread to northern regions, which lead to decreasing diversity from the tropics to higher latitudes (Economo, Narula, Friedman, Weiser, & Guénard, 2018; Ricklefs, 2005). The hypothesis was first used to explain the LDG of angiosperm trees, but then obtained supports from many other taxa (birds: Hawkins & Porter, 2003; mammals: Buckley et al., 2010; ants: Economo et al., 2018). Later on, studies have extended this theory as the phylogenetic niche conservatism (PNC hereafter) hypothesis, so that it can also explain the converse latitudinal patterns of clades originated from temperate zones (e.g., Pyron & Burbrink, 2009; Romdal et al., 2013). It is now generally accepted that geographic diversity patterns are shaped by contemporary environment gradient and historical factors together; the current challenge is to integrate these hypotheses based on ecological and historical processes (Fernández et al., 2016; Hawkins & DeVries, 2009).

Meanwhile, there were still hypotheses focusing on other factors. First, some researchers suggest that the overlapping of species ranges is an important mechanism leading to hump‐shaped latitudinal and altitudinal diversity gradient, which work together with environmental gradient (Colwell et al., 2016; Colwell & Hurtt, 1994; Wang & Fang, 2012). Second, species richness has generally been observed to increase with area, and this relationship has been once viewed as one of the few “rules” in ecology (Lomolino, 2000). Third, the habitat heterogeneity hypothesis proposes that regions with higher habitat heterogeneity can provide more niches for coexisting of more species (Hurlbert, 2004). In addition, human disturbance has strongly altered global ecosystems especially in the past century and thus may be an important factor modifying geographic diversity patterns (Wang et al., 2011). Clearly, the above‐mentioned hypotheses may not be exclusive, and many studies have shown that they worked together in shaping geographic richness patterns (e.g., Wang et al., 2011).

Here, we evaluated these explanations with geographic diversity data of insects. Previous analyses on terrestrial LDG have heavily relied on data from plants and vertebrates (birds and mammals, etc.) (Grimbacher & Stork, 2007). For the most species richness class in the world, insects, LDG has been less well documented. Insecta is an ideal group for examining large‐scale patterns of biodiversity. After four out of five massive extinctions in the Earth's history, insects have retained almost all the orders and have become the most successful animals on the Earth (Diniz‐Filho, Marco, & Hawkins, 2010). Notably, “exceptional” LDG has also been reported for some insect taxa, in addition to many “classical” ones. For example, Hawkins and DeVries (2009) analyzed the spatial diversity patterns of Lepidoptera in the United States and found that species in more basal (i.e., older) subfamilies showed higher richness in the south, whereas more derived (i.e., younger) clades were more species‐rich in the north. Morinière et al. (2016) also found that freshwater arthropods (originated in temperate zone) exhibited high diversity in colder areas, which can be explained by PNC, as the result of an ancestral adaptation to temperate environmental conditions. These diversity patterns may reflect the importance of evolutionary processes on insect LDG. However, most studies on insects were conducted for some specific clades (Economo et al., 2018; Morinière et al., 2016; Owens et al., 2017), the latitudinal pattern and the potential drivers for the insect class as a whole still remains uncertain. Recent phylogenies have resolved taxonomic classifications and timing of insect evolution (Misof et al., 2014), but the analyses and interpretations of the macroecological patterns and macroevolution process for insects are still not satisfied, which may be a major constraint for studies in insect macroecology (Diniz‐Filho et al., 2010). Exploring the latitudinal patterns of both richness and phylogenetic structure, for the insect class, will help to provide a general explanation for global distribution of biodiversity.

In this paper, based on insect data documented in 184 nature reserves across China, we tested the relative importance of contemporary environment and historical process on geographic diversity patterns. China has a great climate gradient and is famous for its rich biodiversity, with significantly different evolutionary history from that in Europe and North American (Ricklefs, 1987). For instance, Europe and North America have experienced more severe species extinctions during historical glaciation than China. Meanwhile, China was also the speciation center for many taxa. Such historical factors are generally accepted to be responsible for the significantly higher biodiversity in China than Europe and North America (Qian & Ricklefs, 2000; Ricklefs, 2005). As a result, China provides an ideal region to test hypotheses on broad‐scale diversity patterns, especially the relative influence of the contemporary environment and historical factors on LDG. Our first goal is to test the relative role of nine hypotheses based on contemporary or historical processes (Table 1) in explaining geographic pattern of insect richness and to examine the difference among evolutionary groups. Secondly, for a better analysis of the evolutionary processes underlying insect richness patterns, we also examined latitudinal patterns of phylogenetic structure using mean root distance (MRD). MRD is calculated from a phylogenetic tree of all species in a region, and higher MRD suggests that the regional fauna/flora are (on an average) evolutionarily younger (Hawkins & DeVries, 2009; Kerr & Currie, 1999). We examined how MRD patterns, for each evolutionary group, were affected by historical and contemporary climate, and other environmental factors. Thirdly, we tested two predictions of the PNC hypothesis as follows: (1) The insect species distribution should contain strong historical signals (Hawkins et al., 2014). The colder climates are generally younger and require more time to develop diversity (Pyron & Burbrink, 2009). Consequently, lower latitudes dominated by more basal (older) clades, while higher latitude occupied by clades that are more evolutionarily derived (younger) (Prediction 1); if LDG was shaped by the filtration of cold tolerance, then this influence on species dispersal should be reflected in the geographic patterns of not only species richness but also phylogenetic structure. Thus, it can be predicted that: (2) Climate factors related to winter coldness should be important correlates of richness and MRD, simultaneously (Prediction 2).

**Table 1:** Hypotheses explaining the geographic diversity gradient of insects and the variables used to test each hypothesis

Hypothesis	Variables (abbreviations)
Contemporary environment hypotheses	
Contemporary climate	
Ambient energy	Potential evapotranspiration (PET)
Water–energy dynamic	Mean annual precipitation (MAP) PET, PET^2^
Productivity	Normalized difference vegetation index (NDVI) Gross primary productivity (GPP)
Other environmental factors	
Area	Area of nature reserves (AREA)
Habitat heterogeneity	Number of plant community types (NCT) Maximum elevation (*E*_max_) Topographical roughness (TR)
Human disturbance	Gross domestic product per km^2^ (GDP) Population (POP)
Historical‐process hypotheses	
Freezing tolerance	Mean temperature of the coldest month (MTCM)
Historical climate	Mean annual precipitation of Last Glacial Maximum (LGM_MAP_) Mean temperature of the coldest month of Last Glacial Maximum (LGM_MTCM_)
Historical climate change	Difference between current and LGM annual mean temperature (T_Anomaly) Difference between current and LGM annual mean precipitation (P_Anomaly)

## 2 MATERIALS AND METHODS

### 2.1 Species diversity data

**Figure 1:**
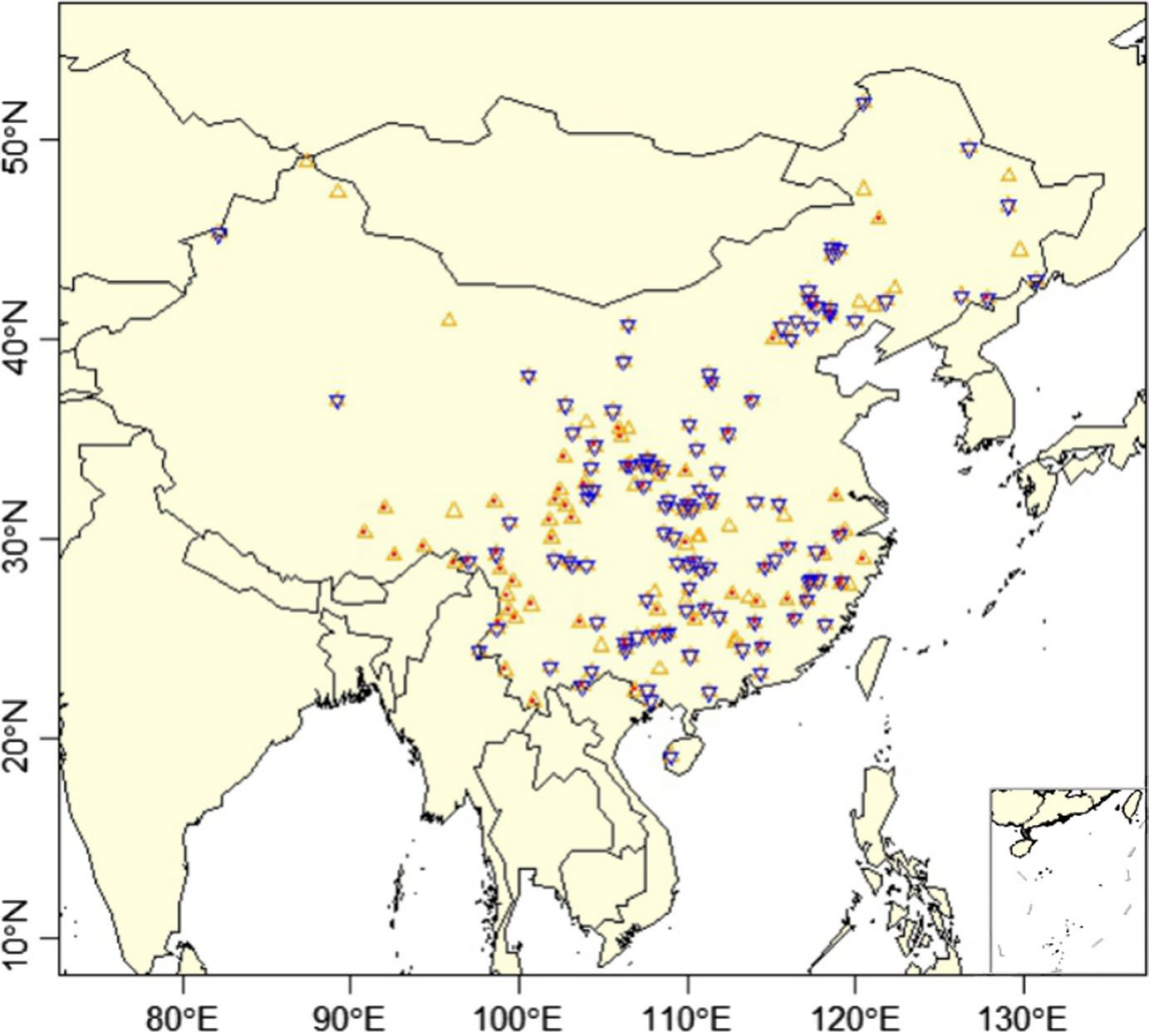
Location of nature reserves across China in this study, in which 184 nature reserves provide overall species richness data (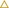), 110 reserves provide insect species lists 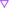), and 80 reserves provide species richness for Palearctic, Oriental, and widespread species (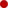)

We compiled a large dataset on insect diversity for 184 nature reserves across China. The data mainly came from the scientific survey reports of the nature reserves (with a few published papers), in which ca. 2/3 of the reports have been published (Appendix S1). These scientific survey reports are vital when the government evaluates whether a site is qualified to be a national nature reserve (Ministry of Ecology and Environment of P. R. China, 1999). Hence, these reports were based on long‐term survey of the nature reserves by experts on local fauna or flora, using the same survey protocol. Further, each scientific report was peer‐reviewed by an expert panel organized by the government, and revisions of the report or supplementary field surveys are needed before the report can pass the scientific evaluation by the experts. Consequently, these scientific survey reports, whether published or not, provided the best available data source for insect diversity of China's nature reserves, at least for large‐scale studies. The nature reserves included in our study spanned a great geographic range from 19°6′ to 51°5′N in latitude and from 82°8′ to 130°4′E in longitude (Figure 1), with an annual mean temperature from −5.9 to 24.7°C and annual mean precipitation from 57 to 2,273 mm. The reserves covered a diverse climate from tropic to boreal across latitudes, and from humid coast to hyperarid central Asia, thus providing a good opportunity to examine the broad‐scale pattern of insect diversity and its potential drivers.

For each nature reserve, we extracted the following data whenever available from the scientific survey reports or literature: (a) total species number for all terrestrial insects; (b) environmental factors, including area and the maximum elevation of the nature reserve, and the number of plant community types (see below for details). These data were available for all the 184 nature reserves. (c) To examine the difference in geographic diversity pattern among evolutionary groups, we also collected the species numbers for different biogeographic affinities [including Oriental, Palearctic, and widespread species; classified based on the world's zoogeographical regions (Holt et al., 2013)], which were available from 80 nature reserves (Figure 1). Studies on plants and animals have shown that different biogeographic affinities revealed rather different geographic diversity patterns, which were closely related to their evolutionary histories (e.g., Hawkins & DeVries, 2009; Owens et al., 2017; Wang et al., 2011). Unfortunately, the scientific reports did not provide the species list for each biogeographic group, which prevent a comparison of latitudinal phylogenetic patterns among groups. (d) To analyze the geographic patterns of MRD, we collected insect species list for each nature reserve, which was available in 110 reserves. We utilized these species lists to categorize insects into another two evolutionary groups. Recent studies have provided a molecular phylogenetic tree resolved to order for insects (Misof et al., 2014). Based on their study, we selected two orders that were originated during a cold period of the Earth's history (Late Carboniferous and Early Permian, 320~274 Ma) as the “cold clades” (see Appendix S2), including Hemiptera (~310 Ma) and Coleoptera (~282 Ma). Meanwhile, another four orders that were originated during a warm geohistorical period (Triassic and Jurassic, 250~145 Ma) were grouped as the “warm clades,” including Hymenoptera (~250 Ma), Orthoptera (~215 Ma), Diptera (~170 Ma), and Lepidoptera (~156 Ma). These six orders are species‐rich and accounted for most of the species (91%) in the species lists we collected. Using these 110 reserves, well distributed across China (Figure 1), we compared the geographic patterns for both species richness and MRD between the warm‐ and cold‐originated clades, for a better test of the PNC hypothesis.

### 2.2 Explanatory variables and the hypotheses tested

Using various explanatory variables, we tested current major hypotheses on geographic diversity patterns based on ecological or historical process (Table 1).

#### 2.2.1 The contemporary climate hypotheses

To test the relationship between insect diversity and contemporary climate, we obtained climate surfaces from the WorldClim database with a spatial resolution of ca. 1 km (Hijmans et al., 2005). We calculated mean monthly temperature and precipitation (means over 1950–2000) across the grids for each nature reserve, using the reserves' digital boundary [mainly obtained from WDPA (World Database on Protected Area)]. Several climate indices were calculated from these monthly temperature and precipitation data: (a) mean annual precipitation (MAP) as an indicator for water availability; (b) potential evapotranspiration (PET), a widely used index of energy availability (O'Brien, 1998); and (c) mean temperature for the coldest month (MTCM), a good surrogate for absolute minimum temperature (Prentice et al., 1992). PET was used to test the ambient energy hypothesis, which suggests energy availability as the major driver of latitudinal richness patterns. MAP and PET together were used to test the water–energy hypothesis, which proposed that water and energy availability were both important (O'Brien, 1993). Considering the possible humped relationship between diversity and energy, PET^2^ was also included in the regression model, following O'Brien (1993). MTCM was used to test the freezing tolerance hypothesis, which was first proposed to explain geographic richness patterns based on ecological processes (see, e.g., Hawkins & Porter, 2003). However, diversity patterns controlled by freezing tolerance are now generally viewed as supports for the PNC hypothesis (e.g., Hawkins et al., 2014). Consequently, here we list this hypothesis as a hypothesis based on historical processes (Table 1).

The productivity hypothesis suggests that climate and other environmental factors affect species richness through productivity (Ruggiero & Kitzberger, 2004). To test this hypothesis, we used the normalized difference vegetation index (NDVI), which is commonly accepted as a good surrogate for vegetation productivity, based on an assumption that insect productivity (and thus insect richness) is limited by vegetation productivity. This is a widely used method when examining the geographic richness patterns of animals, including insects (Hawkins & DeVries, 2009). For each nature reserve, we extracted SPOT NDVI data (with a spatial resolution of 1 km and a temporal resolution of 10 days) between 2005 and 2010 from VITO (http://www.vgt.vito.be) (Maisongrande et al., 2004). For each grid within a nature reserve, monthly NDVI data were obtained by the monthly maximum value composite method according to the standard protocol (Holben, 1986), and then, monthly NDVI data were averaged to obtain annual NDVI. Finally, annual NDVI values were averaged across grids and years as the proxy of vegetation productivity for each nature reserve. In the preliminary analyses, we also calculated the mean gross primary productivity (GPP) between 2005 and 2010 for each reserve, using the same method as NDVI. GPP data (with a spatial resolution of 1‐km) were downloaded from: https://modis.gsfc.nasa.gov/data/dataprod/mod17.php. However, GPP was significantly weaker in explaining insect diversity than NDVI in our study and was highly collinear with some climate factors. Consequently, we used NDVI as the surrogate of vegetation productivity in the final statistical analyses.

#### 2.2.2 Other hypotheses based on the contemporary environment

To test the species–area hypothesis, we documented the area for each nature reserve from the scientific survey reports. To test the habitat heterogeneity hypothesis, we collected the following variables for each nature reserve: the maximum elevation (*E*_max_), the elevational range (ER), and the number of vegetation types (NVT) and number of plant community types (NCT). Each scientific survey report has documented the vegetation types of the nature reserve (e.g., alpine grassland, or subalpine, temperate, warm‐temperate forests) and the major plant community types within a vegetation type (e.g., *Larix, Abies*, and *Picea* forest in the subalpine forest zone). Higher NVT and NCT clearly can provide more diverse habitats and resources for insects, and previous studies have found a significant relationship between animal richness and the diversity of vegetation (community) type (e.g., Gu et al., 2016). However, NVT was excluded from final data analyses because it was closely related to NCT and less powerful in explaining richness patterns.

*E*_max_ and ER have long been used as surrogates for topographical heterogeneity in previous studies (e.g., Rahbek et al., 2007). However, some studies suggested that the two metrics might be not accurate enough and showed that topographical roughness (TR) could well reflect topographical heterogeneity (Wang & Fang, 2012). We thus used ArcGIS 10.3 and the digital elevation model of each nature reserve (with a spatial resolution of ca. 100 m) (USGS 2006) and calculated TR of each grid with the following formula:

(1) TR=1/Cos([Slope]∗3.14159/180)

where the Slope is the slope of the grid, as calculated from the DEM.

Again, TR was averaged across grids as an indicator of topographical heterogeneity for a nature reserve. In the final data analyses, ER was also excluded to avoid collinearity with *E*_max_.

To test the human disturbance hypothesis, we used the human population (POP) and gross domestic product (GDP) per km^2^ to reflect the relative difference in human disturbance intensity among regions (e.g., Steck & Pautasso, 2008). These two variables were obtained from the GDPGrid_China and PopulationGrid_China datasets, respectively, with a spatial resolution of 1 km (Fu, Jiang, & Huang, 2014; Huang et al., 2014). We used ArcGIS 10.3 to extract the POP and GDP between 2005 and 2010 for each nature reserve and calculated average GDP and POP across grids and years for statistical analyses.

#### 2.2.3 The historical climate hypotheses

Historical climate gradient (e.g., Jansson & Davies, 2008) and climate change (e.g., Sandel et al., 2011) may play a role in explaining large‐scale richness patterns, suggesting the influence of historical processes. To test these hypotheses, we obtained temperature and precipitation in the Last Glacial Maximum (LGM, ~21,000 years BP) from WorldClim (http://www.worldclim.org/paleo-climate1). The following variables were used in our analyses: (a) mean annual precipitation of the Last Glacial Maximum (LGM_MAP_) and (b) mean temperature for the coldest month of the Last Glacial Maximum (LGM_MTCM_). These two variables were selected because the PNC hypothesis suggested that the filtering of species traits to tolerate cold and/or arid environment by historical climate gradient is an important contributor to latitudinal richness patterns (e.g., Hawkins et al., 2014; Zanne et al., 2014). (c) We also calculated the difference in mean annual temperature and precipitation between the Last Glacial Maximum and the present (T_Anomaly and P_Anomaly, respectively) to reflect the amplitude of Late Quaternary glacial–interglacial climate change (Sandel et al., 2011). These variables were used to test the possible influence of historical climate change on geographic diversity gradients (Ordonez & Svenning, 2015). In the final data analyses, P_Anomaly was excluded because of collinearity with T_Anomaly.

### 2.3 Mean root distance

To test the two predictions derived from the PNC hypothesis, we used the species lists from the 110 nature reserves to calculate mean root distance (MRD). The root distance for each species represents the number of nodes separating a species' order from the base of the phylogenetic tree, which was obtained from an insect molecular phylogeny resolved to order (Misof et al., 2014). We used MRD for all species within a nature reserve as a measure of the level of the evolutionary development (Hawkins & DeVries, 2009). Thus, regions with high MRD values support species that are, on average, from more derived orders. We calculated the MRD for overall species and for the “warm” and “cold clades” mentioned above. Our dataset is a large one (99,387 records for each species in each nature reserve), with a total of ~39,000 species across 110 reserves. Since determining the biogeographic affinity for all the species is very difficult (Shen et al., 2015), in this study we are not able to categorize the species into different biogeographic groups and to examine their MRD patterns, which we decided to remain for future analyses.

### 2.4 Statistical analyses

To test these hypotheses and predictions, we first explained species richness and MRD (for all species and for the evolutionary groups mentioned above) with each of the variables in Table 1 to examine their bivariate relationships. In multivariate analyses, to avoid collinearity among predictors, we dropped unnecessary variables based on Akaike's information criterion (AIC) and excluded predictors with a variance inflation factor (VIF) >10 (Quinn & Keough, 2002). To assess the relative roles of contemporary, historical climates, and other environmental factors in shaping geographic diversity patterns, we used random forest models to calculate the importance value for the variables retained in models. Random forest is a nonparametric machine‐learning statistical method that is ideal for ranking the importance of closely related or interacting predictors (Breiman, 2001), which is a common feature of geographic diversity data. Consequently, random forest is increasingly used in large‐scale biodiversity studies (e.g., Hawkins et al., 2014). In each random forest model, 200 regression trees were generated by bootstrapping the original data (Breiman, 2001), the resulting variable importance was normalized by the maximum variable importance to produce standardized measures of variable importance.

Spatial autocorrelation in geographic diversity data can inflate type I errors in statistical analyses and may lead to bias when evaluating the relative importance of predictor variables (Diniz‐Filho, Bini, & Hawkins, 2003; Lennon, 2000). To account for this problem, we calculated Moran's I index for the residuals of the random forest models for each species group, using the SAM 4.0 software (Rangel et al., 2010; available at: https://www.ecoevol.ufg.br/sam/). The results showed that there were no significant spatial autocorrelations in model residuals (Appendix S3). In this situation, the influence of spatial autocorrelation was very slight and thus the variable importance provided by random forest model is reliable (Hawkins, Diniz‐Filho, Bini, Marco, & Blackburn, 2007; Hawkins & Porter, 2003; Hawkins et al., 2014).

In these analyses, species richness and area were log‐transformed to account for the power–function relationship commonly observed between area and regional species richness. Other variables were remained not transformed because there were no clear indications of a violation of normality and homoscedasticity in model residuals. Statistical analyses were done in R studio (R version 3.4.5), and the variable importance was implemented using the “randomForest” R package.

## 3 RESULTS

### 3.1 Latitudinal gradient of insect richness and mean root distance

**Figure 2:**
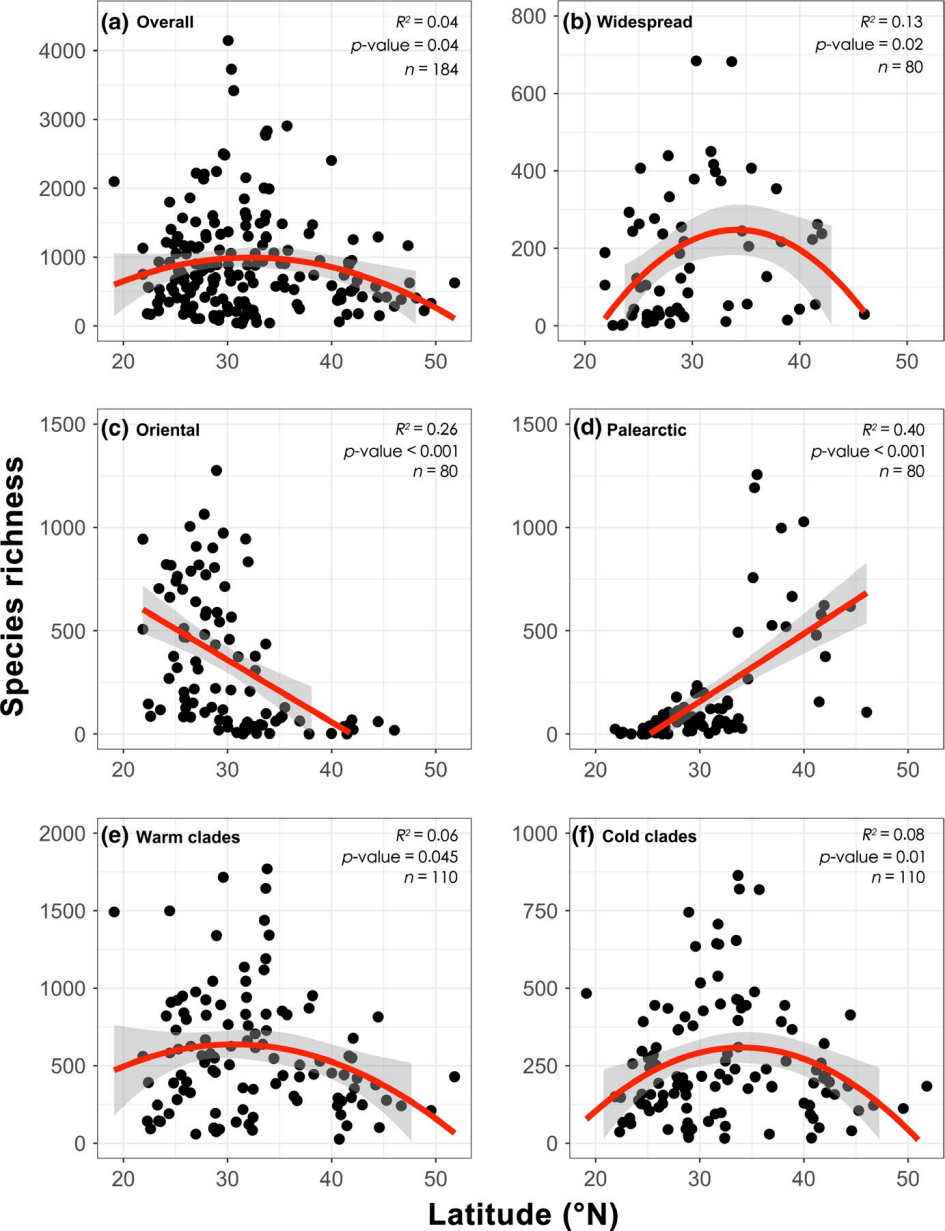
Latitudinal patterns of insect species richness in China's nature reserves for (a) overall species; (b) widespread species; (c) Oriental species; (d) Palearctic species; (e) “warm clades”; and (f) “cold clades” (for details, see Section 2). Regression lines were drawn for relationships significant at *p* < .05. *n*, number of nature reserves

In contrast with the “classical LDG,” the total insect species richness across China's nature reserves did not decrease significantly with higher latitude. Instead, it revealed a hump‐shaped latitudinal pattern, with a richness peak between ca. 30°~35°N (Figure 2a). However, latitudinal richness patterns differed markedly among biogeographic groups: Widespread species showed a humped shape, Oriental species richness decreased significantly with increasing latitude, while Palearctic species exhibited a reverse pattern (Figure 2b–d). This suggests that the range overlapping of different biogeographic affinities at midlatitudes may be an important contributor to the humped diversity pattern of overall species (see Section 4 for details). As for the “warm” and “cold clades,” both of them showed a humped richness pattern (Figure 2e–f). Through fitting the quadratic curves, we obtained that the “warm clades” richness peaked at a lower latitude (31.07°N) than that of “cold clades” (33.92°N), a result in line with the PNC hypothesis.

**Figure 3:**
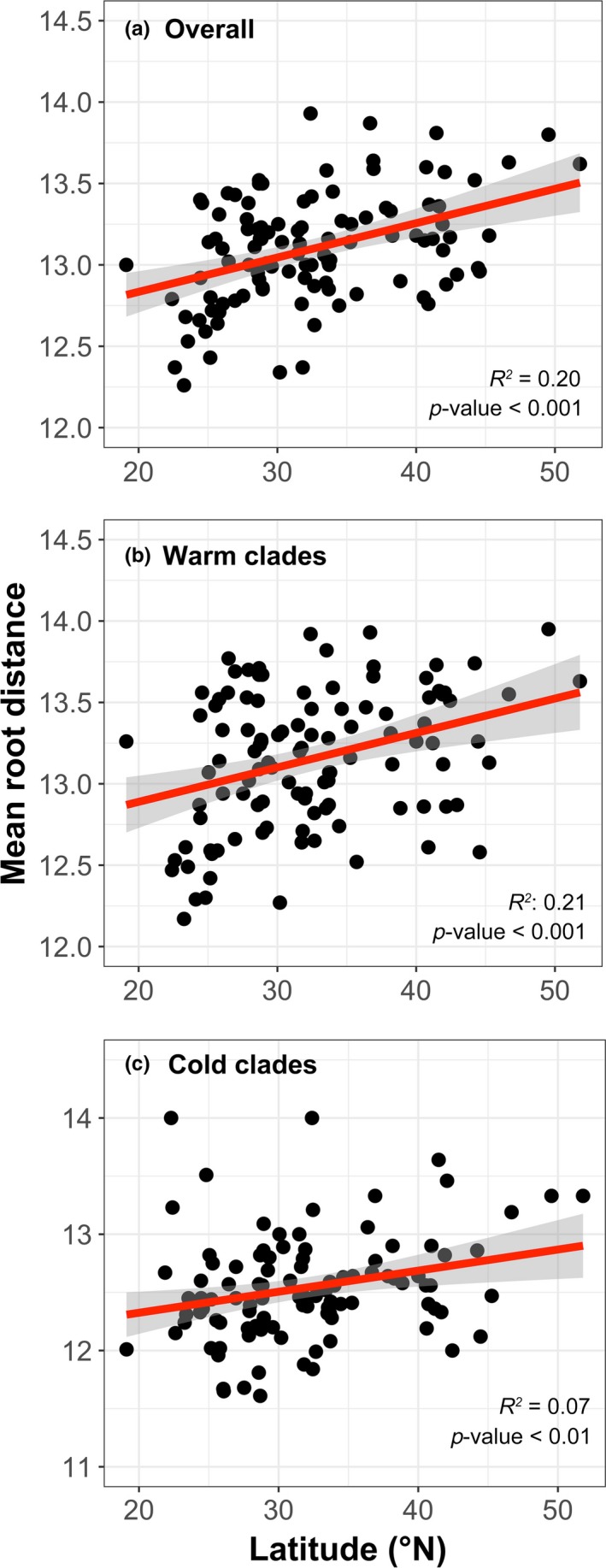
Latitudinal patterns of mean root distance for insects in 110 natural reserves: (a) overall species, (b) “warm clades,” and (c) “cold clades.” Regression lines were drawn for relationships significant at *p* < .05

In support of Prediction 1, MRD for overall species was positively related to latitude (Figure 3), suggesting that insect assemblages at high latitudes are evolutionarily younger than that at low latitudes. The MRD for both “warm” and “cold clades” increased with higher latitude; however, the former clades increased more rapidly (slope = 0.025 and 0.018, respectively). This difference between groups is also consistent with the PNC hypothesis.

### 3.2 Environmental correlates of species richness and mean root distance

In bivariate analyses, historical factors were ranked among the top three strongest correlates of species richness or MRD, for most species groups (in seven out of nine cases) (Table 2). Contemporary climatic factors were also among the strongest correlates for most species groups, but the correlations were generally weaker than that of historical climate (in four out of nine cases). Habitat heterogeneity variables (NCT and *E*_max_) were among the strongest correlates for three species groups, and the correlations were weaker than climate variables. Area and disturbance variable generally did not show strong correlations with richness or MRD.

**Table 2:** Bivariate relationship of species richness and phylogenetic structure (mean root distance) with variables for contemporary factors (climate, area, habitat heterogeneity, and human disturbance) and historical factors. We examined all insects in the nature reserves together, for species with different biogeographic affinities (widespread, Oriental, and Palearctic) and for clades originated in the warm and cold geohistorical periods (“warm” and “cold,” respectively). For each relationship, the percentage of variations explained was reported, with the top three strongest correlates for each clade boldfaced. For abbreviations of the variables, see Table 1 (’*p* < .1; * *p* < .05; ** *p* < .01; *** *p* < .001)

	Species richness						Mean root distance		
	Overall species	Widespread	Oriental	Palearctic	Warm	Cold	Overall	Warm	Cold
Number of reserves	184	80	80	80	110	110	110	110	110
Contemporary climate									
NDVI	9.31***	1.15	18.93***	0.88	9.69***	8.24**	1.54	0.62	2.12’
MAP	7.51***	1.53	48.96***	8.41**	3.45*	0.47	5.28*	2.29’	7.32**
PET	9.20***	1.17	34.28***	9.29**	1.54	0.37	9.53***	6.03**	5.15*
PET^2^	7.46***	1.11	27.62***	10.93**	1.65	0.17	7.70**	5.88**	3.41*
Area									
Ln(AREA)	0.28	1.62	0.91	1.99	0.50	0.22	0.67	0.92	3.34*
Habitat heterogeneity									
*E*_max_	6.83**	**3.81’**	2.67	0.54	5.26**	6.71**	1.94’	0.54	2.48’
NCT	6.71**	1.47	6.85*	1.84	8.77**	9.25**	0.46	0.09	0.82
TR	2.44*	0.77	1.23	3.59’	2.87*	5.68**	0.97	0.35	0.20
Disturbance									
GDP	0.83	0.35	0.58	0.09	1.01	0.03	0.06	1.02	0.14
POP	16.5***	2.03	18.64**	1.24	7.16*	5.27’	0.82	1.44	1.18
History									
MTCM	1.03	**4.49’**	41.75***	29.68***	0.96	0.34	14.17***	9.53***	7.88**
LGM_MAP_	6.14**	1.66	45.97***	9.99**	3.22*	0.11	4.43*	1.82’	7.15**
LGM_MTCM_	2.82*	5.17*	43.2***	27.76***	0.34	0.42	15.18***	10.87***	8.16**
T_Anomaly	7.77***	1.36	13.92***	0.96	2.19’	1.16	7.77**	6.23**	4.13*

Multivariate analyses showed that historical climatic variables (especially LGM_MTCM_) played a key role in explaining insect richness patterns (Table 3). LGM_MTCM_ was among the strongest predictors for most evolutionary groups (variable importance >0.87), and MTCM and T_Anomaly were important for widespread species. Notably, LGM_MTCM_ was generally also the strongest predictor of MRD, consistent with our Prediction 2 that winter coldness should be important predictors for both species richness and MRD. Contemporary climatic factors were ranked among the strongest predictors of richness and MRD in only three out of nine models, and their variable importance was generally much lower than historical climate. These results suggest that contemporary climate supplements historical processes in shaping geographic diversity patterns.

**Table 3:** Relative importance for contemporary environmental factors and historical factors in explaining geographic diversity and phylogenetic patterns of insects in nature reserves across China, as obtained with random forest models. Variable importance >0.8 was boldfaced

	Species richness						Mean root distance		
	Overall species	Widespread	Oriental	Palearctic	Warm	Cold	Overall	Warm	Cold
Contemporary climate									
NDVI	0.05			0.60					
MAP	0.07		**0.87**				0.69	0.07	**0.86**
PET	0.23				0.53	0.35	**1.00**	0.50	**0.98**
PET^2^									
Area									
Ln(AREA)	0.05		0.15	0.20	0.34	0.78	0.22	0.08	
Habitat heterogeneity									
*E*_max_	**1.00**	0.78		0.11	**0.95**	**1.00**	0.18	0.07	**0.99**
NCT	0.16	**1.00**		0.14	0.22	0.53			
TR	0.18		0.46				0.17	0.01	0.09
Disturbance									
GDP	0.21		0.11	0.41	0.26	0.71			
POP	0.35	0.67							
History									
MTCM		**0.83**							
LGM_MAP_		0.18							
LGM_MTCM_	0.32		**1.00**	**1.00**	**1.00**	**0.87**	**0.98**	**1.00**	**1.00**
T_Anomaly		**0.82**							

While human disturbance variables (POP and GDP) and area were retained in many models, their variable importance was commonly low (medium in a few cases). Interestingly, measures of habitat heterogeneity (*E*_max_ and NCT) also presented to be strong predictors for many species groups (overall, widespread, “warm” and “cold” clades) (Table 3).

## 4 DISCUSSION

Understanding the mechanisms underlying LDG is essential for biodiversity conservation under a rapid changing world (Chown & Gaston, 2000). Here, we used insect data across China's nature reserves, together with various environmental factors, to evaluate the relative importance of different processes in shaping LDG. While many animal and plant taxa exhibit increasing species richness toward the equator, we found that overall insect richness revealed a hump‐shaped latitudinal pattern in China. Our results invite the interpretation that niche conservatism and range overlapping are at least among the critical mechanisms involved in generating contemporary insect diversity gradient.

### 4.1 Phylogenetic niche conservatism

While geographic patterns of species distribution are clearly affected by contemporary climate gradients, it is increasingly recognized that species distributions are also strongly governed by ancestral climatic affinities (Romdal et al., 2013). Our results are consistent with this idea and provided several supports to the PNC hypothesis as follows.

First, we found that insect richness revealed converse latitudinal patterns between biogeographic groups (Figure 2b,c). The decrease in richness for Oriental species, which originated in warm climate, can be well explained by the tropical conservatism hypothesis (see Introduction). Our data also showed that Palearctic species, originated from the temperate zone, had higher richness in the north. This is consistent with recent studies, which also found increased diversity at higher latitude, and suggested that the inverse latitudinal patterns may result from that these clades originated from temperate zone and have retained their ancestor's climatic niche (Morinière et al., 2016; Visser et al., 2014). Thus, the contrasting latitudinal patterns of Oriental and Palearctic species in our study both lend support to the existence of niche conservatism, which provide a unified explanation for why species are more difficult to colonize areas with greater climatic difference from their ancestors. In addition, the “cradle” zone of a clade also has more evolutionary time to accumulate higher diversity; this mechanism can further contribute to the decreasing species numbers away from their “cradle” (Buckley et al., 2010).

Secondly, our analyses showed that historical climatic factors (especially LGM_MTCM_) have the strongest influence on not only richness but also phylogenetic structure (MRD), for all evolutionary groups (Table 3). This suggests that the underlying mechanism is that relevant ecological traits (e.g., cold tolerance) of niche conservatism may have limited dispersal of clades between temperate and tropical areas (Pyron & Burbrink, 2009). Interestingly, we found that winter coldness is important for LDG of both Oriental and Palearctic species. While the decrease in Oriental species richness is clearly because fewer species can survive the harsher winter at higher latitude, the converse LDG of Palearctic species seems not to be completely explained by that these species cannot tolerate the warmer winter at lower latitude. Studies have shown that many temperate species can survive the climates in the south, but a major obstacle for their southward dispersion is that they present to be weaker competitors and are usually outcompeted by those clades originated from warmer regions (e.g., Fragniere, Bétrisey, Cardinaux, Stoffel, & Kozlowski, 2015). Our results support this idea, because the correlation of Palearctic species LDG with winter temperature may be partly caused by the increase in competition due to more Oriental species at lower latitude (which was controlled by winter coldness). Another obstacle for the southward dispersion of temperate species is that some insects may need specific climate condition to complete their life history. For instance, in the United States some butterfly groups are species richer in temperate zone, because the cooler winter in mountains and forests there is more suitable for their diapause (Hawkins & DeVries, 2009). Thus, while ecological processes (e.g., competition) clearly also contributed to geographic diversity patterns, they had to work based on the heritages of historical processes.

Thirdly, the geographic patterns of phylogenetic structure for insect assemblages are also consistent with the predictions of PNC hypothesis. Previous studies have shown that MRD increased with higher latitude for some insect taxa (e.g., Hawkins & DeVries, 2009); here, we demonstrated this is also true when examining the insect class as a whole (Figure 3a). We also found that the MRD for “warm clades” increased more rapidly along latitudinal gradient than that of the “cold clades,” suggesting that the insect orders that were originated during a warm historical climate have experienced stronger filtering since the global cooling initiated at the end of the Eocene (Ricklefs, 2005; Appendix S2).

### 4.2 The role of range overlapping

The overall insect species richness across China revealed a hump‐shaped latitudinal pattern (Figure 2), instead of a classical monotonous decreasing one. Many studies have shown that the overlapping of species ranges is an important mechanism leading to hump‐shaped latitudinal and altitudinal patterns, which work together with environmental gradient (Colwell et al., 2016; Wang & Fang, 2012). Our data support this idea because of reasons as follows. (a) Overall species richness peaked between 30~35°N, a latitudinal zone with the Hengduan Mountain Ranges in the west, Qinling Mountain Ranges in middle, and the Huaihe Rivers in the east. These mountains and rivers were not only the boundary between temperate and subtropical climate in China, but also the boundary between Palearctic and Oriental biogeographic regions in East Asia (He et al., 2017; Holt et al., 2013). In another word, the 30~35°N zone can accommodate not only many Oriental species originated from a warmer climate, but also Palearctic species originated from temperate zone (Figure 2c,d). Further, widespread species richness also peaked here (Figure 2b). Consequently, we suggest that the range overlapping of different biogeographic affinities between 30~35°N is an important contributor to the humped latitudinal gradient of overall insect richness. (b) Studies have repeatedly suggested the Hengduan Mountain Ranges as a speciation center in East Asia, and the Qinling Mountain Ranges were also considered as an important species dispersal corridor during historical climate change (Norton, Jin, Wang, & Zhang, 2011). Thus, the dispersal of species northward or southward in this region can further increase the overlapping of species ranges, which also contribute to the hump‐shaped latitudinal patterns (He et al., 2017). (c) It is noteworthy that many other taxa in China also revealed a richness peak in regions around the Hengduan and Qinling Mountains, including birds, angiosperms, and gymnosperms (Ding et al., 2006; Feng, 2008; Li et al., 2009). These studies again support the key role of range overlapping of different fauna (flora) at midlatitudes in shaping the latitudinal diversity patterns in China. (d) Similar to these studies, our results showed that the insect richness of “warm” and “cold clades” also peaked around 30~35°N (Figure 2e,f). Thus, it seems that humped latitudinal diversity patterns are common in China, instead of an exception. Each of the “warm” and “cold clades” has included species from the Palearctic, Oriental, and widespread biogeographic groups (Shen et al., 2015). Consequently, we believe that the humped latitudinal patterns of “warm” and “cold clades” were also contributed by the range overlapping mechanisms mentioned above.

### 4.3 Contemporary climate and habitat heterogeneity

Contemporary climate has long been proposed to play a leading role in shaping the latitudinal patterns of biodiversity (Hawkins & Porter, 2003; O'Brien, 1998). However, we found that contemporary climate is generally not very important in explaining insect diversity patterns in China, when historical climate and habitat heterogeneity were considered simultaneously in the models (Table 3). On the other hand, contemporary climatic factors related to productivity (such as MAP, NDVI, PET) did enter the richness models for most clades (Table 3). Thus, our result is consistent with the species pool hypothesis, which suggests that while regional species pools were mainly determined by historical processes, the ecological processes driven by productivity are responsible for the further filtering of regional fauna into actual species pool (Romdal et al., 2013). In another word, species diversity patterns are shaped by historical and ecological processes together.

Habitat heterogeneity is well known to be important for richness patterns at local to regional scales (Jansson & Davies, 2008; Visser et al., 2014). Our study at a broad scale also found that *E*_max_ revealed a high importance (which was comparable to that of historical climate) in models for some species groups (Table 3). A nature reserve with higher *E*_max_ can accommodate more species because of reasons as follows. (a) Higher mountains generally lead to higher topographic heterogeneity (Hurlbert, 2004), which provides more diverse micro‐environment for climate and other resources. However, this seems not a major driver in our study because topographic roughness, a more direct measure of topographic heterogeneity, was not a strong predictor. (b) High mountains had great altitudinal climate gradient and thus can accommodate diverse species (Janzen, 1967; Rahbek et al., 2007), which may be an important contributor in our study because *E*_max_ differed greatly among natural reserves (603 ~ 6,624 m). (c) High mountains are also related to many evolutionary processes (Qian & Ricklefs, 2000). For instance, high mountains are often found to be speciation center as a result of the island effect (Kattan & Franco, 2004). Meanwhile, high altitudes are well known to be refugia for temperate species during the glacial age (Owens et al., 2017). Thus, the high explanatory power of *E*_max_ in our study may have also included the effect of historical processes, which requires further examination.

## 5 CONCLUSION

Our study provides insight into the mystery of how the most species‐rich invertebrate class diversity is exhibited on a broad scale. We suggest niche conservatism and range overlapping as major contributor to the hump‐shaped latitudinal gradient of total insect species richness, while habitat heterogeneity and contemporary climate played a second role. Area and human disturbance seem not strong predictors for broad‐scale patterns of insect richness, at least in China. If we had analyzed only the overall species richness pattern, we were not able to identify the contrasting latitudinal patterns (Figure 2) and the importance of historical climate for each evolutionary group (Table 3), and thus might have overlooked the critical roles of niche conservatism and range overlapping. Clearly, examining different evolutionary groups is necessary for exploring the underlying mechanisms for geographic diversity patterns. It can help us to understand the origin and maintenance of global diversity patterns, which is critical for biodiversity conservation in an era of rapid global change.

## CONFLICT OF INTEREST

The authors declare no competing interests.

## AUTHOR CONTRIBUTIONS

Yueming Lyu collected and analyzed the data, and wrote the manuscript. Xiangping Wang designed the study and wrote the manuscript. Juchun Luo provided most of the data sources and revised the manuscript.

## Data Availability

Data used in the analyses are available at: https://doi.org/10.5061/dryad.msbcc2fth.
