# Supplementary material for: Geographic patterns of insect diversity across China's nature reserves: The roles of niche conservatism and range overlapping
Source: Ecol Evol. 2020 Mar 14;10(7):3305–17. doi: 10.1002/ece3.6097 (PMC7141035; doi:10.1002/ece3.6097)

**Appendix 1** Source of insect diversity data included in this study.

**Published books and papers:**

1. Chen, F., Feng, Z. L., Cui, G. F., Huang, D. H., & Shi, J. (2013). Research on Insect Fauna in Qingshan Natural Reserve, Inner Mongolia. *Chinese Agricultural Science Bulletin,* **29**, 87-91.
2. Chen, J., Ou, X. H., Liu, B., Zhao, Y. S., Guo, C. C., Liu, Q., Li, S. Q. & Hu, S. H. (2006). Insect resource and diversity of Yuanjiang Nature Reserve in the dry-hot valley of Yunnan. Advances in Biodiversity Conservation and Research in China VII: Proceedings of the Seventh National Symposium on the Conservation and Sustainable Use of Biodiversity in China.
3. Cheng, J. M. (2013). Scientific survey report of Liupanshan National Nature Reserve. Beijing, Science Press.
4. Cheng, Y. Q. (2007). Study on Insect communities and diversity in Kongtong Mountains. *Journal of Gansu Forestry Science and Technology,* **32**, 4-9.
5. College, S. F. (1995). Gaoligongshan National Nature Reserve. Beijing, China Forestry Publishing House.
6. Cui, G. F., & Xing., S. H. (2009). Scientific survey report of Beijing Labagoumen Natural Reserve. Beijing, China Forestry Publishing House.
7. Dang, K. L. (2006). Scientific survey report and studies on biodiversity of Shaanxi Guanyinshan Natural Reserve. Beijing, China Forestry Publishing House.
8. Deng, H. P. (2015). Biodiversity of Chongqing Dabashan National Nature Reserve. Beijing, Science Press.
9. Department of Guizhou Province Forestry. (2000a). Scientific survey report of the Fodingshan Nature Reserve in Guizhou. Beijing, China Forestry Publishing House.
10. Department of Guizhou Province Forestry. (2000b). Scientific survey report of the Zhujiashan Nature Reserve in Guizhou. Beijing, China Forestry Publishing House.
11. Gao, E. H., Wang, Z. X., & Wang, Z. C. (2012). Scientific Investigation and Research on Duheyuan Nature Reserve in Hubei Province. Beijing, Science Press.
12. Guo, Y. R. (2010). Scientific survey report of Yangjifeng Nature Reserve in Jiangxi Province. Beijing, Science Press.
13. Guo. C., Yuan, S. X., Liu, F., & Ren, B. Z. (2012). Preliminary study on Insect diversity in Heilihe National Nature Reserve. *Journal of Jilin Agricultural University,* **34**, 31-36.
14. He, Z., Yang, D. D., Tong, X. W., & Li, Y. Y. (2007a). Species diversity of Insect in Shunhuangshan Nature Reserve. *Journal of Zhejiang Forestry Science & Technology*, **27**, 24-29.
15. He, Z., Yang, D. D., Tong, X. W., Wang, B. L., & Gu, Z. R. (2007b). Species diversity of Insects in Badagongshan National Nature Reserve of Hunan Province. *Journal of Central South University of Forestry & Technology,* **27**, 61-65.
16. He, Z., Yang, W. F., Tong, X. W., Huang, J. H., & Wei, M. C. (2011). Investigation and analysis on insect resources in Nanyue Hengshan National Nature Reserve, Hunan Province. *Hunan Forestry Science & Technology,* **38**, 1-5.
17. Hong, Q. P. (2007). Investigation and Research on Natural Resources of Fengyang Mountain. Beijing, China Forestry Publishing House.
18. Huang, R. X., Jiang, T., Liu, J. P., Wu, W., Ouyang, T., Ji, Y., A, E. T., Kang, J. X. & Nuer, B. (2004). The Insect’s Fauna of the Two Rivers (Ertix River and Ulungur River) Springhead Protection Area in Altay Mountains. *Journal of Xinjiang University (Natural Science Edition),* **21**, 399-406.
19. Li, C. L. Xu., Z. H., Song, Y., Zhang, X. M. Situ, Y. X., & Mo, F. Y. (2014). Analysis on Insect Fauna in Nangun River Nature Reserve in Yunnan Province. *Journal of Southwest Forestry University,* **34**, 82-86.
20. Li, S. G., & Zhang, F. (2014). Biodiversity and Conservation Management of Pangquangou National Nature Reserve in Shanxi. Beijing, China Forestry Publishing House.
21. Li, W. Y. (2001). Distribution and Fauna analysis of Insect resources in Haifeng Nature Reserve of Zhanyi County. *Forest Inventory and Planning,* **26**, 58-61.
22. Li, Z. J. (2009). Scientific survey report of Jiulingshan Nature Reserve in Jiangxi Province. Beijing, Science Press.
23. Li, Z. J. (2012). Biodiversity Research of Taining World Natural Heritage Site. Beijing, Science Press.
24. Li, Z. J. (2013). Study on Biodiversity of Jiangxi Wuyuan Forest Bird Nature Reserve. Beijing, Science Press.
25. Liu, R. L. (2013). Scientific survey report of the Jiangxi Ganjiangyuan National Nature Reserve. Beijing, China Forestry Publishing House.
26. Liu, W. B., & Duan, H. M. (2008). Analysis of Biodiversity Flora of Taizi Mountains Nature Reserve. *Journal of Gansu Forestry Science and Technology,* **33**, 20-23.
27. Liu, X. M., Guo, Y. R., & Liu, R. L. (2010). Scientific survey report of Jiangxi Qiyunshan Natural Reserve. Beijing, China Forestry Publishing House.
28. Liu, X. Z. (2002). Scientific survey and Forest Ecosystem Research of Jiangxi Jiulianshan Nature Reserve. Beijing, China Forestry Publishing House.
29. Liu, X. Z. (2005). Scientific survey report of the Jiangxi Guanshan Natural Reserve. Beijing, China Forestry Publishing House.
30. Liu, X. Z. (2006). Scientific Investigation and Research on Rare plant species Community in Matoushan Nature Reserve, Jiangxi Province. Beijing, China Forestry Publishing House.
31. Liu, X. Z., & Fang, F. S. (2001). Scientific survey report of Jiangxi Wuyishan Nature Reserve. Beijing, China Forestry Publishing House.
32. Liu, X. Z., & Wang, L. (2010). Investigation and Research on Biodiversity of Lushan Nature Reserve in Jiangxi Province. Beijing, Science Press.
33. Lu, L. (2005). Preliminary investigation report on Insect resources of Guangxi Jinzhongshan Nature Reserve. *Forest Resources Management,* **6**, 69-73.
34. Ning, S. J., Tan, X. F., & Su, Y. (2010). Key Biodiversity Area: Scientific survey report of Jiuwanshan Nature Reserve in Guangxi Province. Beijing, Science Press.
35. Qi, J. W., Huang, Z. J., & Dai, Y. (2012). Studies on biodiversity of Hunan Liubuxi National Natural Reserve. Beijing, China Forestry Publishing House.
36. Ren, Y. (2006). Studies on biodiversity and management of Taibaishan Nature Reserve. Beijing, China Forestry Publishing House.
37. Ren, Y. (2008). Scientific survey report of Micangshan Nature Reserve in Shaanxi Province. Beijing, Science Press.
38. Song, C. S. (1994). Scientific survey report of the Funiushan Nature Reserve. Beijing, China Forestry Publishing House.
39. Song, C. S. (1999). Scientific survey report of the Hubei Houhe Natural Reserve. Beijing, China Forestry Publishing House.
40. Song, J. X. (2009). Preliminary study on Insect diversity of Tongbiguan Nature Reserve in Yunnan. *Forest Inventory and Planning,* **34**, 17-22.
41. Tang, X. Q., & Lu, J. (2011). Diversity and Faunal Analysis of Known Insect Species in Cibagou National Nature Reserve. *Journal of Mountain Agriculture and Biology,* **30**, 487-491.
42. Wang, K. F., Wen, Z. Q., & Feng, Q. J. (2014). Scientific survey report of Taibai Niuwei River Nature Reserve in Shaanxi Province. Beijing, Science Press.
43. Wang, L., Tian, R. X., Lyu, Z. B., Wang, X. Z., & Wang, H. J. (2015). Study on the Insect Fauna of the Anxi Extra-arid Desert National Nature Reserve. *Forestry Science & Technology,* **40**, 52-56.
44. Wang, Z. X. (2013). Study on Biodiversity and Protection of Hubei Nanhe Nature Reserve. Beijing, Science Press.
45. Wu, H. (1995). Insects of Baishanzu in Eastern China. Beijing, China Forestry Publishing House.
46. Wu, Y. F. (2007). Scientific Investigation and study on biodiversity of Liaoheyuan Nature Reserve in Hebei Province. Beijing, Science Press.
47. Wu, Y. F., Zhao, J. C., & Cheng, J. (2006). Investigation and research on biodiversity of Maojingba Nature Reserve in Hebei Province. Beijing, Science Press.
48. Xing, S. H. (2013). Scientific surbey of Beijing Wulingshan Nature Reserve. Beijing, China Forestry Publishing House.
49. Xu, H. C., Wu, H., Yang, S. Z., Zhao, Y. M., & Ying, Z. H. (2002). Insect species diversity of Mount Tianmu in China. *Journal of Zhejiang Forestry College,* **19**, 350-355.
50. Xu, Z. H. (2013). Insects resource of Xiaowutai Mountain. Beijing, China Forestry Publishing House.
51. Yang, L. Y. (2006). Study on Insect resources in Xishuangbanna Nature Reserve. *Forest Inventory and Planning,* **31**, 49-53.
52. Ye, Y. Z., & Li, P. X. (2014). Scientific survey report of Jigongshan National Nature Reserve in Henan Province. Beijing, Science Press.
53. Ye, Y. Z., Wang, W. S., & Li, H. S. (2004). Scientific survey report of Xiaoqinling Nature Reserve in Henan Province. Beijing, Science Press.
54. Ye, Y. Z., Zhuo, W. H., & Zheng, X. X. (2012). Scientific survey report of Henan Dabieshan Nature Reserve. Beijing, Science Press.
55. You, Q., Hou, Y. R., & Lu, G. D. (2008). Research state of animal taxonomy fauna and diversity in Maoershan National Nature Reserve in Guangxi. *Guangxi Forestry Science,* **37**, 194-196.
56. Zhang, H. H., Wang, Z. M., & Zhang, X. (2007). Studies on Biodiversity of Leigongshan National Nature Reserve. Guizhou, Guizhou Science and Technology Publishing House.
57. Zhang, S. L., Zhang, X. J., & Yong, S. P. (2013). Scientific survey report of Wulanba Nature Reserve. Beijing, China Forestry Publishing House.
58. Zheng, B. J., & Zhao, M. (2016). Studies on Biodiversity of Eerguna National Nature Reserve. Beijing, Science Press.
59. Zhu, J. (2013). Scientific survey report of Badashan Nature Reserve of Pan County, Guizhou Province. Beijing, China Forestry Publishing House.
60. Zhu, Z. Q. (1999). Scientific survey report of Shennongjia Nature Reserve. Beijing, China Forestry Publishing House.
61. Tan, W. F., & Luo, H. T. (2010). The study and protect biodiversity in Guangxi Dayaoshan Nature Reserve. Beijing, China Environmental Science Press.
62. Lin, P. (2003). Scientific survey report of Fujian Daiyunshan National Nature Reserve. Fujian, Xiamen University Press.
63. Zhu, T. A. (1994). Insects and macrofungi in Zhejiang Gutian Mountain. Zhejiang, Zhejiang Science and Technology publishing House.
64. Wang, X. M. (2011). Scientific survey report of Ningxia Helanshan National Nature Reserve. Ningxia, Sunshine Press.
65. Gao, X. B., & Kang, Y. X. (2007). Scientific survey report of Shaanxi Huangguanshan National Nature Reserve. Shaanxi, Shaanxi Science and Technology publishing House.
66. Dang, K. L. (2009). Scientific survey report of Shaanxi Huangbaiyuan National Nature Reserve. Shaanxi, Northwest A&F University Press.
67. Zhang, F. C. Yang, X. Z., & Li, D. W. (2008). Scientific survey report of Shaanxi Yanan Huanglongshan *Crossoptilon mantchuricum* National Nature Reserve. Shaanxi, Shaanxi Science and Technology publishing House.
68. Biodiversity Research Center in Shanxi Province. (2014). Scientific survey report of Manghe *Macaca mulatta* National Nature Reserve. Shanxi, Shanxi people's publishing house.
69. Zhao, J. C. (2005). Study on the biodiversity of temperate and warm temperate zone: Scientific survey report in Mulanweichang Nature Reserve. Beijing, Science Press.
70. Li, Z. G., Kang, K. G., & Wu, Z. H. (2008). Comprehensive scientific investigation and biodiversity study of Pingheliang Nature Reserve in Shaanxi Province. Shaanxi, Shaanxi Science and Technology publishing House.
71. Liu, X. Y. (1997). Scientific survey report of Zhejiang Qingliangfeng National Nature Reserve. Beijing, China Forestry Publishing House.
72. Zhang, J. Q. (2008). Scientific survey report of Guangdong Shimentai National Nature Reserve. Hubei, Huazhong University of Science and Technology Press.
73. Du, L. H. (2012). Scientific survey report of Beijing Songshan National Nature Reserve. Beijing, China Forestry Publishing House.
74. Li, J. J., & Xie, M. Y. (2001). Scientific survey report of Gansu Taitong-Kongtong Mountain National Nature Reserve. Beijing, China Forestry Publishing House.
75. Li, Z. G., Dang, K. L., & Li, D. W. (2005). Scientific survey report of Shaanxi Tianhuashan National Nature Reserve. Shaanxi, Shaanxi Science and Technology publishing House.
76. Yang, Y. M., Tian, K., & He, S. J. (2008). Scientific survey report of Wenshan National Nature Reserve. Beijing, Science Press.
77. Kang, Y. X., Gao, X. B., & Zhang, X. P. (2006). Scientific survey report of Wuliangshan National Nature Reserve. Shaanxi, Shaanxi Science and Technology publishing House.
78. Chen, S. J. (2006). Scientific survey report of Xinjiang Xiaerxili National Nature Reserve. Xinjiang, Xinjiang Science and Technology publishing House.
79. Hu, J. Z. (2003). A report of the comprehensive survey on Xiaozhaizi Nature Reserve in Sichuan, China. Sichuan, Sichuan Science and Technology publishing House.
80. Liu, S. X., & Qu, J. P. (2002). Scientific survey report of Hubei Xingdoushan National Nature Reserve. Hubei, Hubei Science and Technology publishing House.
81. Hu, J. Z. (2003). A report of the comprehensive survey on Yele Nature Reserve in Sichuan, China. Sichuan, Sichuan Science and Technology publishing House.
82. Yuan, Z. F. (1992). Study on Yingtaogou Nature Protection Experimental in Beijing. Beijing, China Forestry Publishing House.
83. Zhang, J. Q. (2009). Scientific survey report of Guangdong Yunkaishan National Nature Reserve. Hubei, Huazhong University of Science and Technology Press.
84. Wang, S. X. (2007). Biological Resources in Changbai Mountain. Shenyang, Liaoning Science and Technology publishing House.

**Unpublished scientific survey reports:**

1. Academy of Forestry Investigation and Planning. (2008). Scientific survey report of Gansu Chagangliang National Nature Reserve.
2. Administration of the Haitang Mountains Nature Reserve in Liaoning Province. (2002). Scientific survey report of Liaoning Haitangshan National Nature Reserve.
3. Administration of the Qilian Mountain Nature Reserve in Gansu Province. (2005). Qilianshan National Nature Reserve in Gansu Province.
4. Administration of the survey and protect the wildlife in Sichuan Province. (1999). Scientific survey report of Sichuan Wanglang National Nature Reserve.
5. Administration of the survey and protect the wildlife in Sichuan Province. (2005). Scientific survey report of Sichuang Daxiaolangou National Nature Reserve.
6. Administration of the Touersantan Nature Reserve in Gansu Provicne. (2003). Scientific survey report of Gansu Touersantan National Nature Reserve.
7. Central South University of Forestry and Technology. (2006). Scientific survey report of Hunan Jiemuxi National Nature Reserve.
8. Chinese Research Academy of Environmental Sciences. (2005). Scientific survey report of Gansu Guiqingshan National Nature Reserve.
9. College of Biological Sciences and Biotechnology of Beijing Forestry University. (2003). Scientific survey report of Hebei Saihanba Nature Reserve.
10. College of Biological Sciences of Hunan Normal University. (2003). Scientific survey report of Hunan Wuyunjije National Nature Reserve.
11. College of Biological Sciences of Northwest Normal University. (2003). Scientific survey report of Gansu Liancheng National Nature Reserve.
12. College of Biological Sciences of Sichuan University. (2003). Scientific survey report of Sichuan Meigu Dafengding National Nature Reserve.
13. College of Biological Sciences of Sichuan University. (2003). Scientific survey report of Sichuan Xuebaoding National Nature Reserve.
14. College of Biological Sciences of Sichuan University. (2007). Scientific survey report of Sichuan Laojunshan National Nature Reserve.
15. College of Biological Sciences of Sichuan University. (2008). Scientific survey report of Sichuan Heizhugou National Nature Reserve.
16. College of Biological Sciences of Southwest University. (2002). Scientific survey report of Chaqingsongduo National Nature Reserve.
17. College of Environment and resource of Shanxi University. (2009). Scientific survey report of Shanxi Heichashan National Nature Reserve.
18. College of Wildlife Resource of Northeast Forestry University. (2005). Scientific survey report of Heilongjiang Shengshan National Nature Reserve.
19. Forestry Survey and Design Institute of Guangxi. (2005). The comprehensive investigation report of Guangxi Cenwanglaoshan Natural Reserve.
20. Forestry Survey and Design Institute of Guangxi. (2005). The comprehensive investigation report of Guangxi Longtan Natural Reserve.
21. Forestry Survey and Design Institute of Guangxi. (2005). The comprehensive investigation report of Guangxi Shiwandashan Natural Reserve.
22. Forestry Survey and Design Institute of Guangxi. (2007). The comprehensive investigation report of Guangxi Yachang Orchids Natural Reserve.
23. Forestry Survey and Design Institute of Hunan Province. (2003). Scientific survey report of Hunan Huangsang National Nature Reserve.
24. Forestry Survey and Design Institute of Jilin Province. (2003). Scientific survey report of Jilin Hunchun Panthera tigris National Nature Reserve.
25. Forestry Survey and Design Institute of Jilin Province. (2006). Scientific survey report of Jilin Hani National Nature Reserve.
26. Forestry survey and design Institute of Tibet autonomous region. (2000). Scientific survey report of Tibet Cibagou National Nature Reserve.
27. Forestry survey and design Institute of Tibet autonomous region. (2000). Scientific survey report of Tibet Mangkang Rhinopithecus bieti National Nature Reserve.
28. Forestry survey and design Institute of Tibet autonomous region. (2001). Scientific survey report of Tibet Leiwuqi National Nature Reserve.
29. Forestry Survey and Planning Institute in Central and Southern of State Forestry Administration. (2000). Scientific survey report of Guangxi Damingshan National Nature Reserve.
30. Forestry Survey and Planning Institute in Central and Southern of State Forestry Administration. (2006). Scientific survey report of Hunan Gaowangjie National Nature Reserve.
31. Forestry Survey and Planning Institute in the Northwestern of State Forestry Administration. (2007). Scientific survey report of Shaanxi Tianzhushan National Nature Reserve.
32. Guangxi Institute of Botany,Chinese Academy of Sciences. (2002). Scientific survey report of Guangxi Yuanbaoshan National Nature Reserve.
33. Hunan Academy of forestry. (2003). Scientific survey report of Hunan Yingzuiije National Nature Reserve.
34. Inner Mongolia Normal University. (2005). Scientific survey report of Inner Mongolia Gurigesitai National Nature Reserve.
35. Institute of Protect for wildlife in Central South University of Forestry and Technology. (2002). Scientific survey report of Hunan Yangmingshan National Nature Reserve.
36. Institute of Resources and environment of Hebei Normal University. (2008). Scientific survey report of Hebei Qingyazhai National Nature Reserve.
37. Jishou University. (2010). Scientific survey report of Hunan Baiyunshan National Nature Reserve.
38. Research Group of Dafengbao Nature Reserve in Chongqing. (2005). The report of science survey for Dafengbao Nature Reserve, chongqing municipality.
39. Research Group of Karst Forest in Mulun Nature Reserve. (1995). Scientific survey report of Mulun Karst Forest National Nature Reserve.
40. Research Group of Longchi Nature Reserve in Shaanxi Province. (2007). Comprehensive scientific investigation report on Shaanxi longchi Nature Reserve.
41. Research Group of Saiwudang Nature Reserve in Hubei. (2008). Hubei Saiwudang Nature Reserve scientific survey.
42. Research Group of Xuebaoshan Nature Reserve in Chongqing. (2004). Scientific survey report of Chongqing Xuebaoshan National Nature Reserve.
43. Saihanwula Nature Reserve in Inner Mongolia. (1998). Scientific survey report of Inner Mongolia Saihanwula National Nature Reserve.
44. Southwest Forestry University. (1999). Daweishan Nature Reserve.
45. Southwest University. (2009). Scientific survey report of Chongqing Yintiaoling National Nature Reserve.
46. Survey and Planning Institute of State Forestry Administration. (2008). Scientific survey report of Heilongjiang Langxiang National Nature Reserve.
47. The forestry administration of Guangxi. (2005). Scientific survey report of Guangxi Chongzuo Trachypithecus poliocephalus National Nature Reserve.
48. The forestry administration of Hainan Bawangling. (2001). Scientific survey report of Hainan Bawangling National Nature Reserve.
49. Yuan, Z. K., & Zhu, D. A. (2007). Scientific survey report of Hunan Kanglong National Nature Reserve.

**Appendix 2** Based on the study of Misof *et al*. (2014), and the Geologic Temperature Record (*Wikipedia*, [http://en.wikipedia.org/wiki/Geologic_ temperature_record](http://en.wikipedia.org/wiki/Geologic_%20temperature_record)), we selected two insect orders that were originated during a cold period of the earth’s history (Late Carboniferous and Early Permian, 320 ~ 274 Ma, see the blue transparent area) as the “cold clades”: Hemiptera (~310 Ma) and Coleoptera (~282 Ma). Meanwhile, another four orders that were originated during a warm geohistorical period (Triassic and Jurassic, 250~145 Ma, see the yellow transparent area) were grouped as the “warm clades”, including Hymenoptera (~250 Ma), Orthoptera (~215 Ma), Diptera (~170 Ma), and Lepidoptera (~156 Ma). The first insect order (Protura) was originated during a warm historical climate, and the whole insect class have experienced strong filtering by coldness since the global cooling initiated at the end of the Eocene.


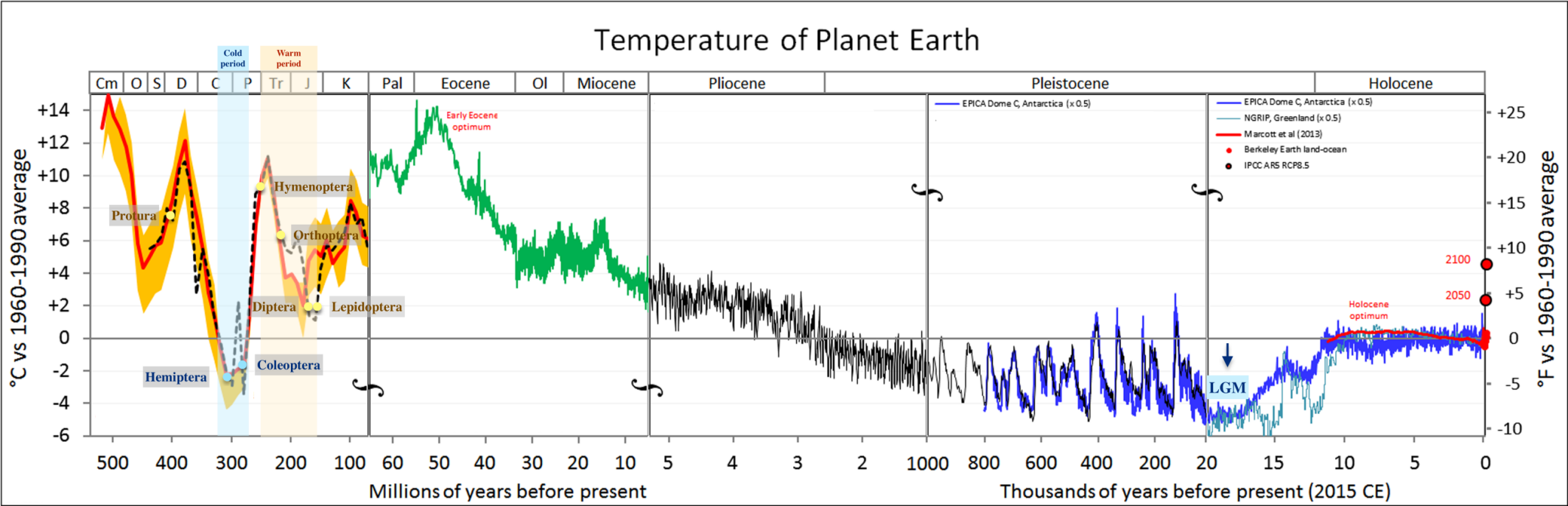


**Appendix 3** Correlograms of Moran’s I showing patterns of spatial autocorrelation for the residuals of random forest models in Table 3. Filled circles indicate there were no significant spatial autocorrelation (p > 0.05).

**(1) Species richness models**


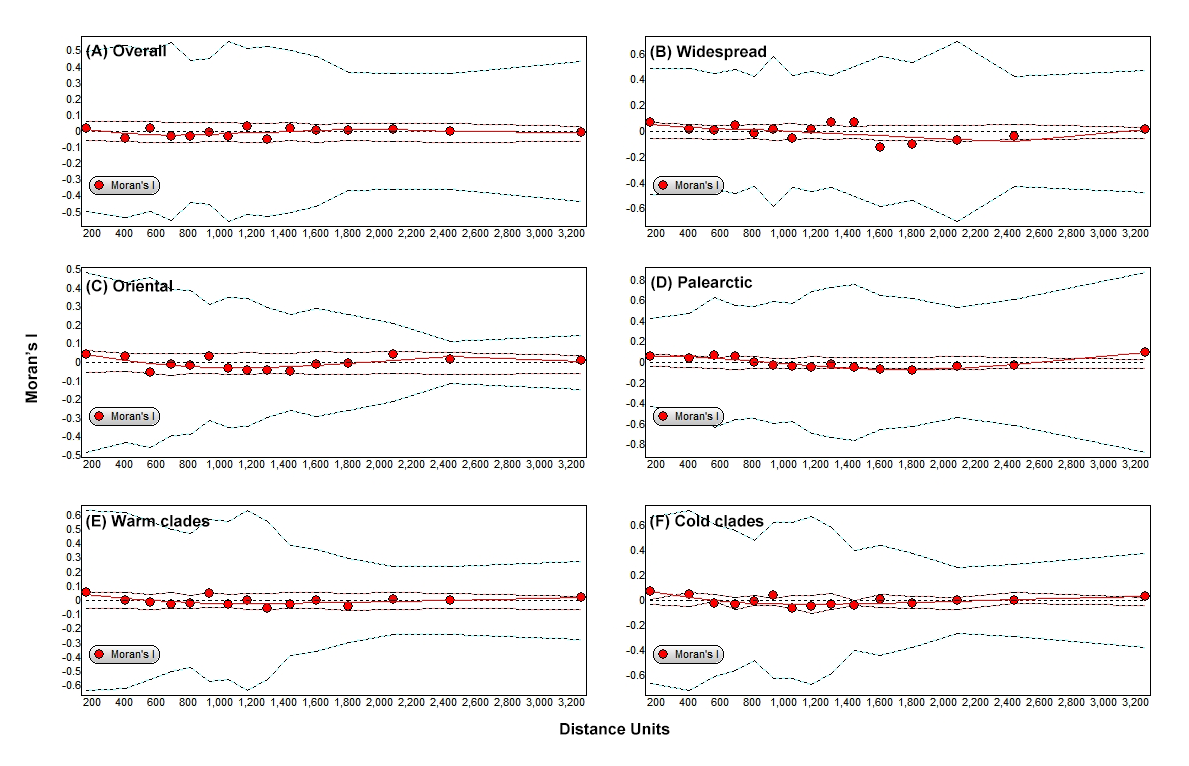


**(2) Mean root distance models**


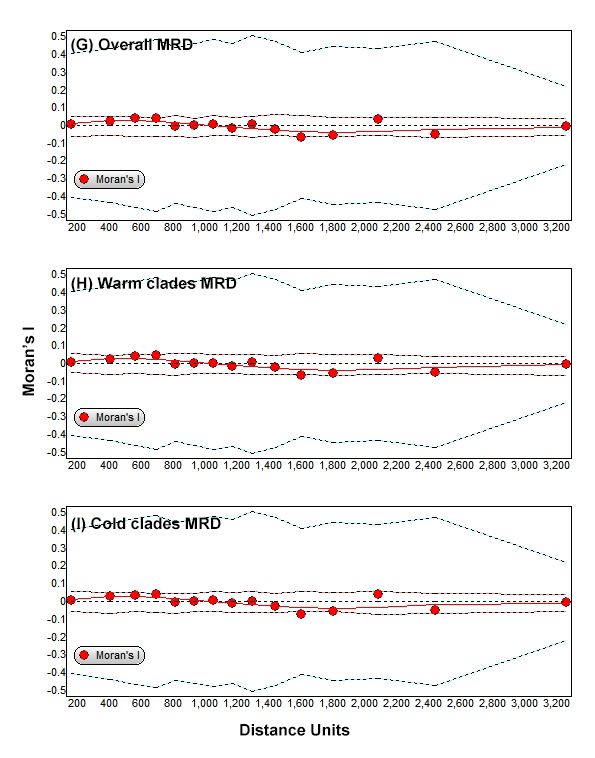

Supplement: Supplementary file 1 [file ECE3-10-3305-s001.docx]
